# The complete chloroplast genome of *Salvia liguliloba* Y. Z. Sun (Lamiaceae)

**DOI:** 10.1080/23802359.2022.2097486

**Published:** 2022-07-25

**Authors:** Zewei Du, Fuhai Yuan, Yanbo Huang, Yue Chen, Ying Cheng, Yukun Wei, Dongfeng Yang

**Affiliations:** a Key Laboratory of Plant Secondary Metabolism and Regulation in Zhejiang Province, College of Life Sciences and Medicine, Zhejiang Sci-Tech University, Hangzhou, China; b Changzhou Menghe Shuangfeng Chinese Herbal Medicine Technology Co. Ltd., Changzhou, China; c Shanghai Key Laboratory of Plant Functional Genomics and Resources, Shanghai Chenshan Botanical Garden, Shanghai Chenshan Plant Science Research Center, Chinese Academy of Sciences, Shanghai, China

**Keywords:** *Salvia liguliloba*, chloroplast genome, phylogenetic analysis

## Abstract

*Salvia liguliloba* Y. Z. Sun is a plant species endemic to the Tianmu Mountains. In this study, we assembled the complete chloroplast genome of *S. liguliloba*. The chloroplast genome of *S. liguliloba* was 151,490 bp with quadripartite structure in length, which contained 124 encoded genes, including 79 protein-coding genes, eight ribosomal RNA genes, and 37 transfer RNA genes. Our phylogenetic analysis result based on 54 chloroplast genomes revealed that *S. liguliloba* was closely related to *S. miltiorrhiza* according to the current sampling extent in Lamiaceae.

The genus *Salvia* encompasses more than 980 species and is the largest genus in the angiosperm family Lamiaceae (Will and Claßen-Bockhoff 2017; Hu et al. 2018). *Salvia liguliloba* Y. Z. Sun (1935) is one of the genus Salvia, a plant species endemic to the Tianmu Mountains, and is only distributed in Zhenjiang Province, China. *Salvia liguliloba* has a small corolla, short filament, two stamens with sterile and united lower thecae (Huang et al. 2015). As a Chinese herbal remedy, *S. liguliloba* was used to treat hematemesis, metrorrhagia, dysentery with bloody stools, and traumatic bleeding (Ran et al. 1993). However, the complete chloroplast (cp) genome sequence of *S. liguliloba* has not been reported so far. In this study, its cp genome was successfully assembled and annotated, and its relationship with closely related species was investigated.

The *S. liguliloba* individual was collected from Lin’an, Zhejiang, China (GPS: 30°23′45.00″N, 119°28′35.31″E, voucher S0747, contact person name: Yukun Wei, Email: ykwei76@hotmail.com). The specimen and extracted DNA were deposited at the Herbarium of Shanghai Chenshan Botanical Garden (CSH). The DNA Plantzol Reagent was used to extract DNA from its leaf (Invitrogen, Carlsbad, CA, USA). The Illumina platform was used to produce the raw data (Illumina Inc., San Diego, CA, USA). All research reported in this paper has been conducted ethically and responsibly and is in full compliance with all relevant codes of experimentation and legislation. Ethical approval has been obtained from the appropriate local ethics committee or Institutional Review Board and where relevant, informed consent has been obtained. With adaptors removed, about 5.72 G high-quality clean reads (150 bp PE read length) were obtained. NOVOPlasty v2.7.2 (Dierckxsens et al. 2016) was used to assemble the complete chloroplast genome of *S. liguliloba* with default settings. GeSeq (Tillich et al. 2017) and Geneious Prime were used for alignments and annotation with *Salvia miltiorrhiza* plastome (GenBank: HF586694) as a reference (Biomatters Ltd., Auckland, New Zealand).

The whole chloroplast genome of *S. liguliloba* (GenBank accession No. MZ855771) is 151,490 base pairs, with a typical circle form and 38.0% GC content. It is composed of a large single-copy region (LSC with 82,717 bp, 36.2% GC content), a small single-copy region (SSC with 17,546 bp, 38.6% GC content), and two inverted repeat regions (IR, 25,613 bp, 39.4% GC content). *S. liguliloba* has a total of 133 genes, including 87 protein-coding genes, eight rRNA genes, and 37 tRNA genes. There are seven protein-coding genes (*rps12*, *rpl2*, *rpl23*, *ycf2*, *ycf15*, *ndhB*, and *rps7*), six tRNA genes (*trnI-CAU*, *trnL-CAA*, *trnV-GAC*, *trnI-GAU*, *trnA-UGC*, and *trnR-ACG*) and all four rRNA genes (*rrn*16, *rrn*23, *rrn*4.5, and *rrn*5) have two copies. Six protein-coding genes (*rps16*, *atpF*, *rpoC1*, *petB*, *petD*, and *rpl16*) have one intron each and five (*ndhB*, *rpl2*, *ndhA*, *ycf3*, and *clpP*) have two introns.

Fifty-four species of Lamiaceae with accessible complete chloroplast genomes were chosen to confirm the phylogenetic position of *S. liguliloba* (Figure 1). The complete cp sequences were aligned using MAFFT version 1.3 (Katoh and Standley 2013). The maximum likelihood (ML) phylogenetic analyses were constructed using the program IQ-TREE (Nguyen et al. 2015) with 5000 bootstrap repetitions under the TVM + F + R3 model. Phylogenetic research revealed that *S. liguliloba* was closely related to *S. miltiorrhiza* according to the current sampling extent.

**Figure 1.**
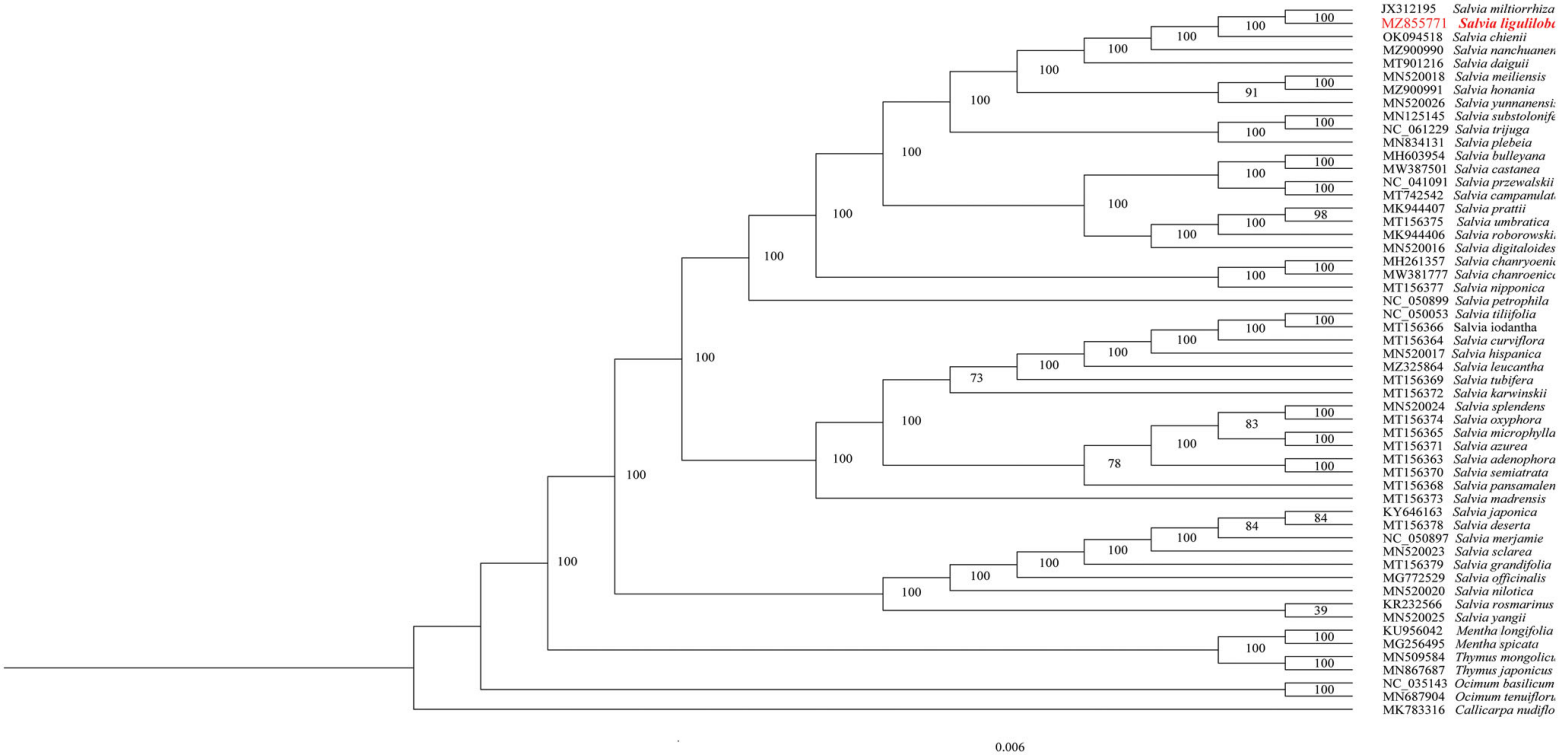
Phylogenetic relationship of *Salvia liguliloba* in Lamiaceae using maximum likelihood (ML) method based on 54 species complete chloroplast genomes (accession numbers were listed behind each taxon. Statistical support values were shown on nodes).

## Data Availability

The data that support the findings of this study are openly available in GenBank of NCBI at https://www.ncbi.nlm.nih.gov, reference number MZ855771. Raw Illumina data is available at the Sequence Read Archive (SRA) under accession SRR15533979. BioProject (PRJNA756452), BioSample accession: SAMN20866468.
